# Supplementary material for: Genomes of the dinoflagellate Polarella glacialis encode tandemly repeated single-exon genes with adaptive functions
Source: BMC Biol. 2020 May 24;18:56. doi: 10.1186/s12915-020-00782-8 (PMC7245778; doi:10.1186/s12915-020-00782-8)
Supplement: Supplementary file 2 — Additional file 2: Supplementary Figures 1 through 5. [file 12915_2020_782_MOESM2_ESM.pdf]

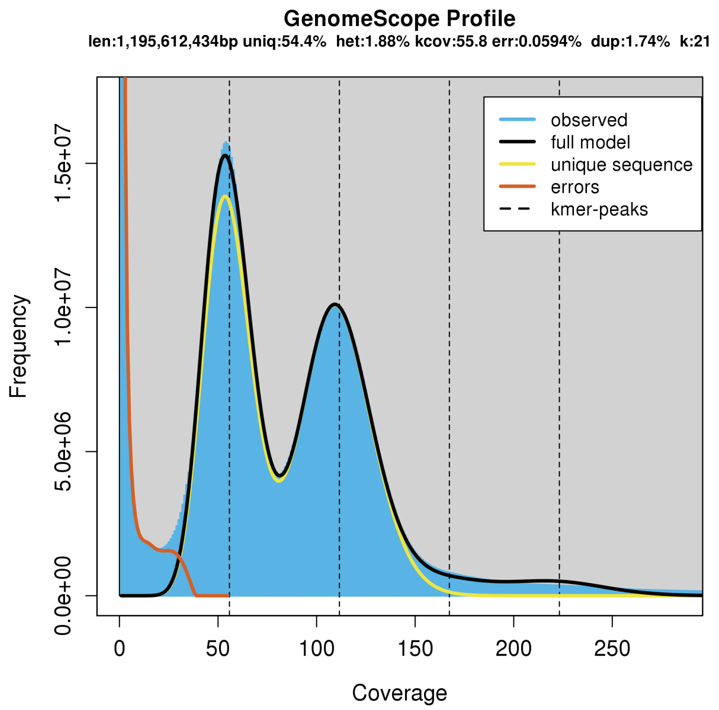

Supplementary Figure 1. GenomeScope 21-mer profile for CCMP2088.

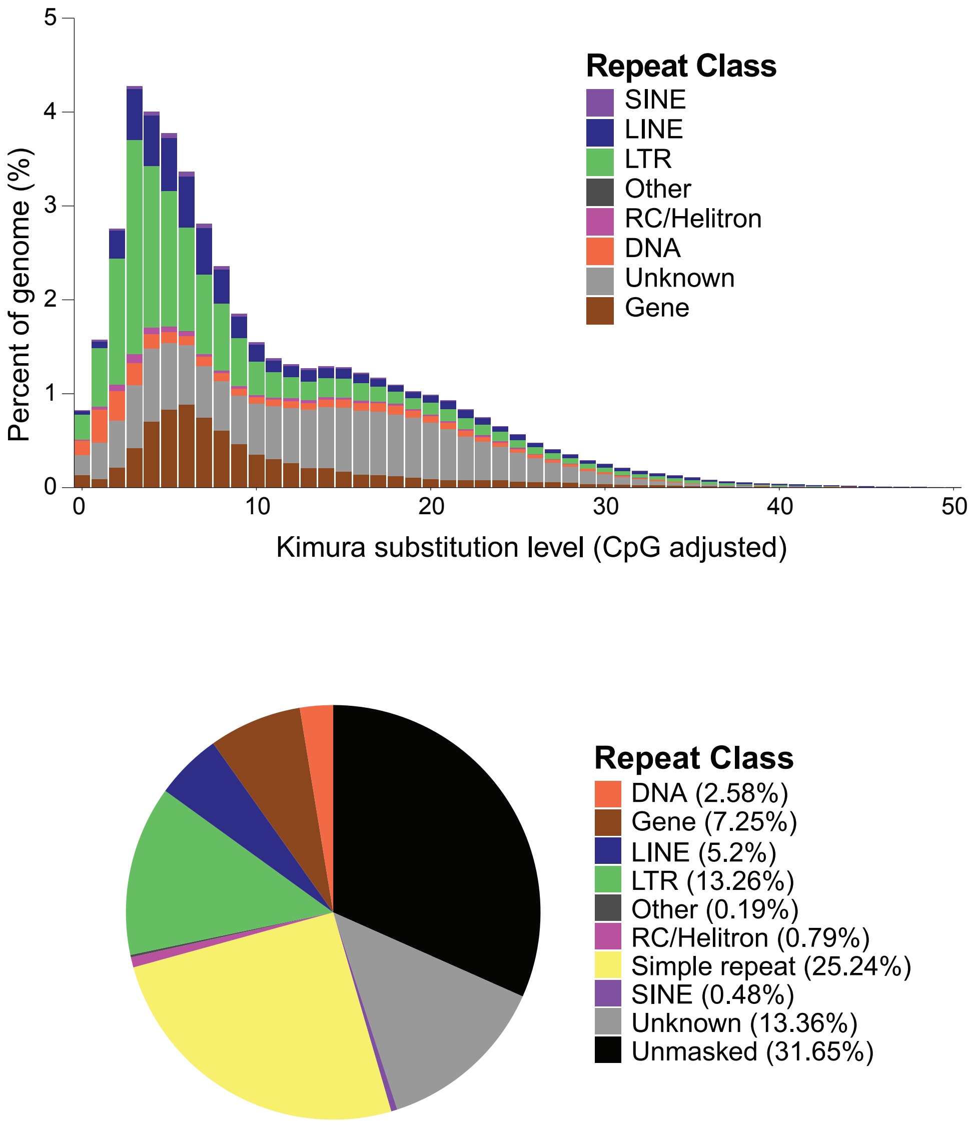

Supplementary Figure 2. Interspersed repeat landscape and proportion of distinct repeat classes in the assembled genome of CCMP2088, relative to sequence divergence in Kimura substitution level.

a

b

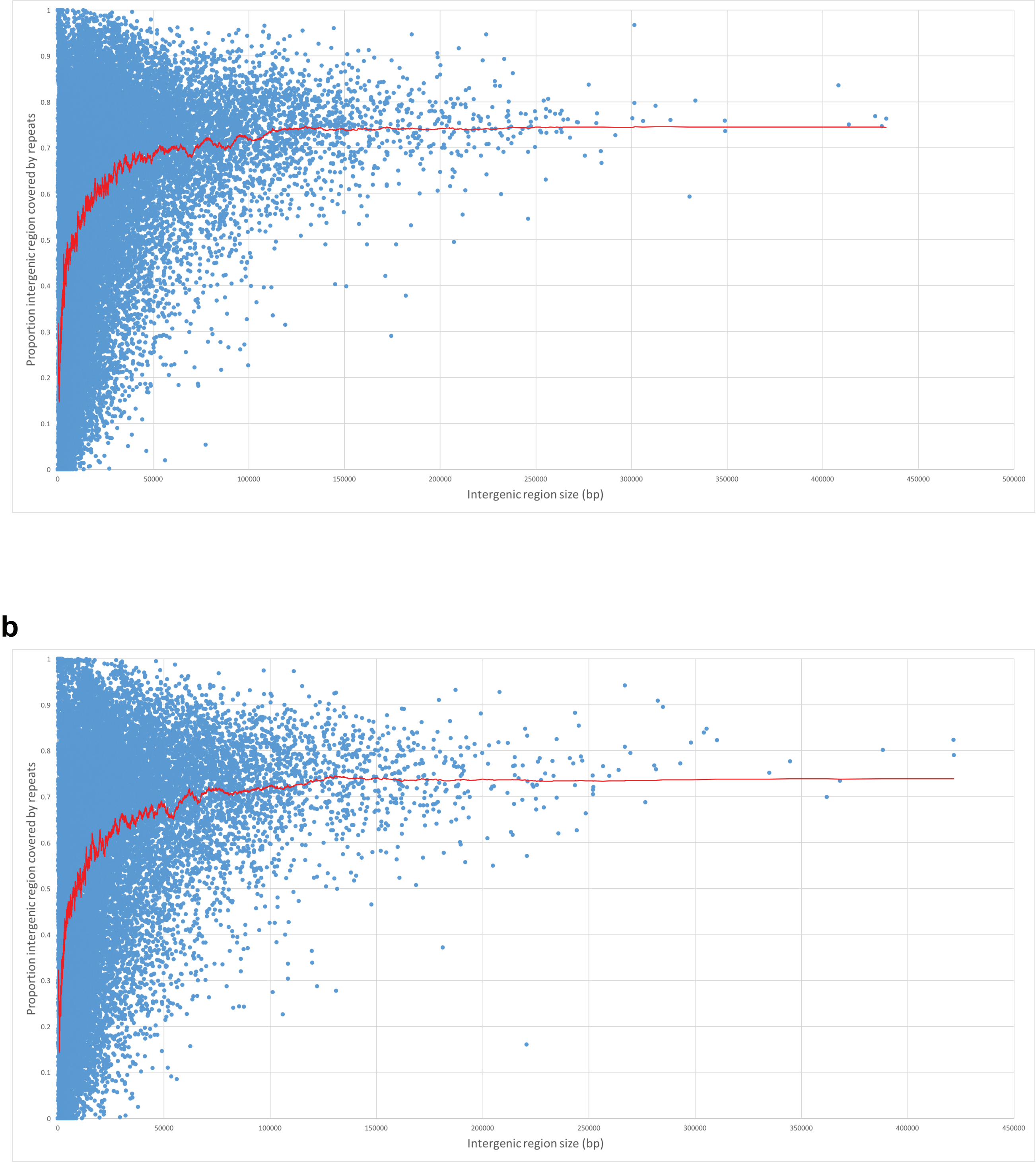

Supplementary Figure 3. Relationship between length of intergenic regions and their coverage by repeats for the predicted genes from (a) CCMP1383 and (b) CCMP2088. The red trend line was constructed using a moving average with a window size of 250.

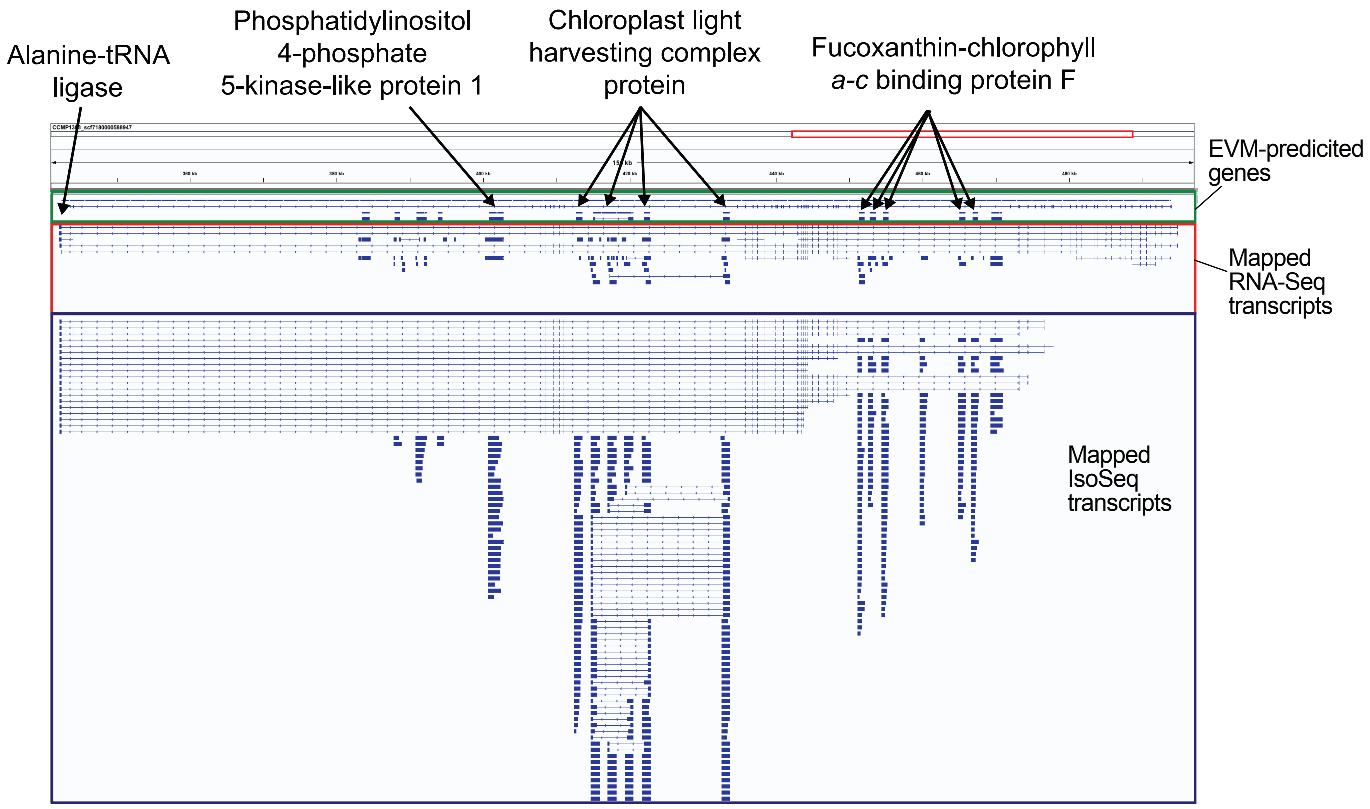

Supplementary Figure 4. An example of a genome region containing genes nested within the long introns of a putative alanine-tRNA ligase (from scaffold CCMP1383_scf7180000588947). The EvidenceModeler predicted genes, mapped IsoSeq transcripts and mapped RNA-Seq transcripts are shown in the green, red and blue boxes.

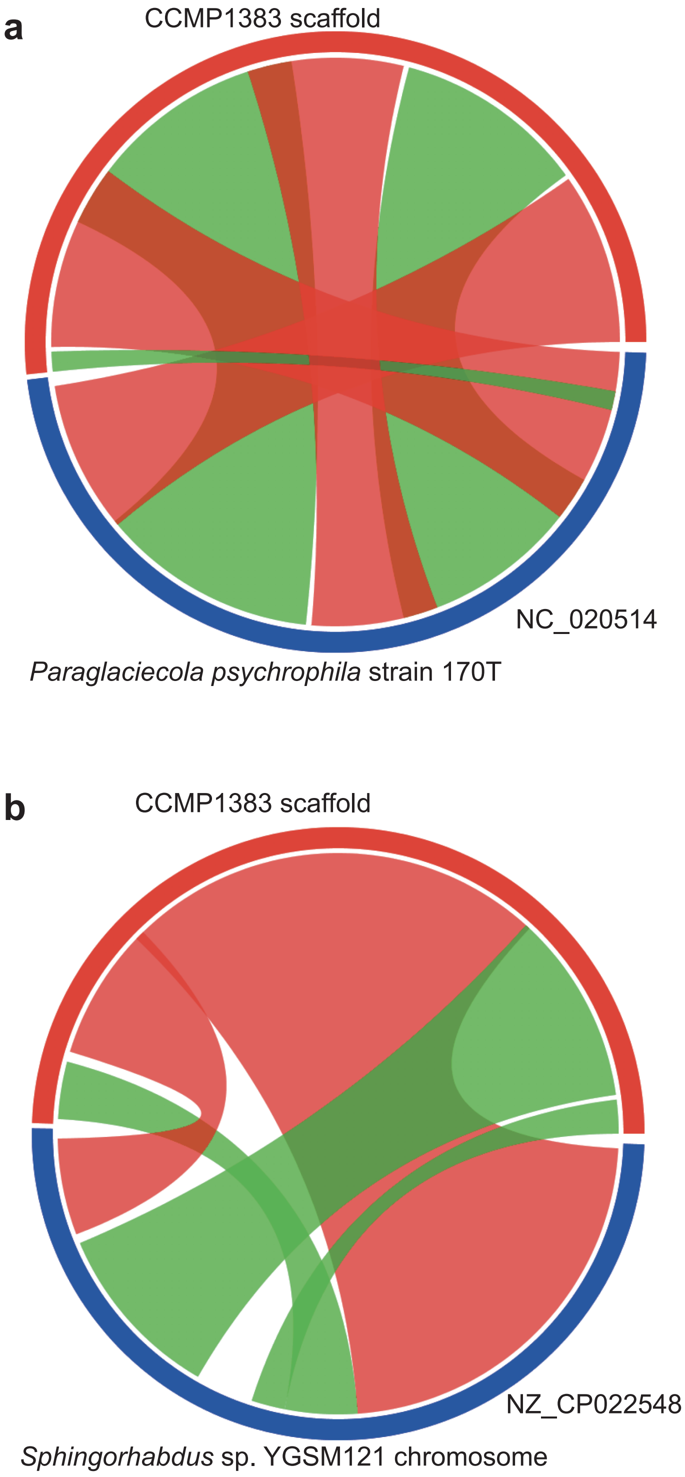

Supplementary Figure 5. Conserved synteny between the two sequenced bacterial scaffolds and the published (a) *Paraglaciecola psychrophila* strain 170T (GenBank NC_020514) and (b) *Sphingorhabdus* sp. YGSM121 (GenBank NZ_CP022548) genomes. Syntenic regions between the two sequences are shown with ribbons; red representing direct and green represents inverted regions.
